# Effect of Topical Timolol on Healing of Immature Breast Scars After Mammoplasty: A Randomized Controlled Trial With Blinded Assessors and Patients

**DOI:** 10.1111/jocd.70261

**Published:** 2025-05-28

**Authors:** Nahid Nafissi, Fatemeh Najafi, Alireza Jafarzadeh, Elham Behrangi, Masoumeh Roohaninasab, Seyyedeh Tahereh Rahimi, Sepideh Salehi, Amir Jafari‐Nasirmahalleh, Nazanin Hoseini, Azadeh Goodarzi

**Affiliations:** ^1^ Department of Surgery, Rasool Akram Medical Complex Research Development School of Medicine, IUMS Tehran Iran; ^2^ Breast Health & Cancer Research Center IUMS Tehran Iran; ^3^ School of Medicine Iran University of Medical Sciences Tehran Iran; ^4^ Department of Dermatology, Rasool Akram Medical Complex Clinical Research Development Center (RCRDC), School of Medicine Iran University of Medical Sciences Tehran Iran; ^5^ Skin and Stem Cell Research Center Tehran University of Medical Sciences Tehran Iran; ^6^ Department of Dentistry Shahid Beheshti University of Medical Sciences Tehran Iran

**Keywords:** clinical trial, immature scar, mammoplasty, postoperative scar, RCT, scar, timolol, topical timolol, wound

## Abstract

**Introduction:**

Wound healing is a complex process encompassing four main stages: hemostasis, inflammation, cell proliferation, maturation, and differentiation. Timolol (TM) may influence these stages, particularly re‐epithelialization. This study aims to evaluate the 1‐month effects of timolol on acute surgical wounds in post‐mammoplasty patients.

**Objectives:**

To investigate the efficacy of topical timolol in improving postoperative breast scars, aiming to guide future treatment protocols and prescriptions.

**Methods:**

A total of 12 patients who underwent bilateral mammoplasty were enrolled in this double‐blind randomized clinical trial. Treatment commenced 48 h post‐surgery; one breast was treated with 0.5% timolol eye drops, while the contralateral breast received distilled water (control). Patients were advised to minimize sun exposure and pressure on the treated area, and no additional oral or topical medications were prescribed. Cleansing with a prescribed cleanser occurred every 3 days. Cosmetic assessments were conducted by a specialist at 10 and 30 days post‐surgery using a 10‐point Likert scale. Data were analyzed using two‐way repeated measures ANOVA.

**Results:**

Timolol significantly reduced erythema over time (Interaction, *p* < 0.0001; Treatment, *p* = 0.02), with an average decrease of 5.38 points (95% CI: 4.22–6.55) compared to 4.41 points (95% CI: 3.83–5) for placebo. The difference in reduction was 0.972 points (95% CI: 0.18–1.7). A significant improvement in the aesthetic appearance of the breast was also noted (Interaction, *p* < 0.0001; Treatment, *p* = 0.015), with timolol enhancing the aesthetic score by approximately 5.5 points (95% CI: 4.9–6.2) versus 4.58 points (95% CI: 3.4–5.7) for the placebo. Overall, timolol improved the aesthetic score by 0.972 points (95% CI: 0.23–1.7) more than the placebo.

**Conclusion:**

Topical application of 0.5% timolol significantly improved the aesthetic appearance and reduced erythema of post‐mammoplasty breast scars over a 1‐month period. The results demonstrate a measurable clinical benefit, with statistically significant differences favoring timolol over placebo. These findings suggest that early intervention with topical timolol may offer a safe, effective, and non‐invasive option for optimizing scar outcomes in surgical patients.

**Abbreviations**
MMPs: Matrix metalloproteinases
TM: Timolol

## 1 Introduction

Recent advancements have refined our understanding of the wound healing process, which involves four distinct yet overlapping phases: hemostasis, inflammation, proliferation, and remodeling. The hemostatic phase begins immediately after injury and is characterized by vasoconstriction and platelet aggregation [1, 2]. This is followed by the inflammatory phase, where neutrophils and macrophages orchestrate the immune response and initiate tissue repair. During the proliferative phase, keratinocytes and fibroblasts contribute to re‐epithelialization, angiogenesis, and extracellular matrix (ECM) formation. Finally, in the remodeling phase, collagen is reorganized, and the scar matures [3, 4]. Studies published between 2023 and 2025 have highlighted the critical roles of adrenergic signaling, oxidative stress modulation, and cytokine profiles in regulating these processes [1, 2, 3, 4].

These insights underscore the importance of targeted topical therapies, such as timolol, in modulating cellular behavior to optimize healing outcomes and reduce hypertrophic scarring.

Keratinocytes not only respond to adrenergic signaling but also produce their own epithelial signals, thus forming a self‐sufficient catecholaminergic system [4]. TM has been demonstrated to modulate the inflammatory response in epithelial‐mediated healing disorders [2]. While the exact mechanism of TM is complex and not fully understood, it has been shown to enhance the regulation of neutrophils and macrophages during the inflammatory phase [2] and may encourage anti‐inflammatory differentiation [5, 6].

Additionally, TM promotes keratinocyte proliferation and migration, primarily through cytoskeletal remodeling and the inhibition of ERK and AKT dephosphorylation. This leads to a 28% increase in keratinocyte migration and a 2.5‐fold increase in ERK phosphorylation, facilitating wound re‐epithelialization [7, 8]. TM also affects fibroblast migration and proliferation by blocking βARs and reducing ERK phosphorylation [2].

Other βAR antagonists have shown similar effects in enhancing wound healing and reducing profibrotic mRNA, thereby improving the aesthetic appearance of scars [1]. A decrease in βAR expression can result in hypertrophic scarring due to uncontrolled fibroblast proliferation. Furthermore, TM increases vascular permeability and VEGF secretion, although the exact mechanisms underlying angiogenesis in wound healing remain unclear [9].

During the maturation phase, TM influences scar formation through 4‐hydroxy‐0′‐deoxyguanosine (8OHdG) and modulates matrix metalloproteinases (MMPs) [7].

Given the significance of effective wound healing and the treatment of immature scars, this study investigates the efficacy of topical timolol in treating immature scars in patients following surgical mammoplasty.

## 2 Methods and Materials

This study employed a randomized controlled trial (RCT) design to investigate the effectiveness of topical timolol for treating immature scars following bilateral mammoplasty. The research population included patients visiting the hospital for this elective surgical procedure.

### 2.1 Participants

The selection was made using a purposive sampling method, whereby one breast of each patient served as the control (saline) and the other as the intervention (timolol). The initial sample size was set at 15 participants for a pilot study due to the lack of similar research. Following pilot results, the final sample size was determined to be 12 individuals.

### 2.2 Inclusion Criteria

Participants were required to be between 18 and 65 years of age, necessitating simultaneous surgical intervention with uniform scar thickness, and presenting with no skin lesions or diseases at the incision site prior to or during the study. Additionally, they should not require any additional treatments (surgery or topical medications) at the surgical site during the study and must not be using any medications that could interact with timolol.

### 2.3 Exclusion Criteria

Individuals with diabetes or severe depression, those who are pregnant or breastfeeding, and participants with unrealistic expectations regarding treatment outcomes were excluded from the study. Additional exclusion criteria included any allergies to materials used in the study, a history of arrhythmia, asthma, myocardial infarction (MI), congestive heart failure (CHF), low blood pressure, bradycardia, or bronchospasm, as well as participation in another clinical study.

### 2.4 Procedure

Prior to the intervention, a comprehensive medical history was obtained, followed by a general and systemic physical examination with a focus on skin assessments. Patients were instructed to refrain from using any topical or systemic treatments that might interact with timolol.

Digital imaging of all treatment areas was conducted from multiple angles. The skin surrounding the surgical sites was assessed for uniformity in observational and tactile characteristics. The suture count and type of thread used were identical in both surgical sites. The surgical areas were initially covered with sterile gauze and petroleum jelly for 48 h.

After the dressing was removed 48 h post‐surgery, patients were instructed to apply five drops of timolol 0.5% eye drops (Timolol Darou Pakhsh 0.5%, 5 mL Ophthalmic Drops) to one breast and five drops of distilled water to the other breast once daily for 4 weeks. Participants received guidance on minimizing sun exposure and avoiding pressure on the treated area. No oral or topical medications were prescribed. Additionally, they were instructed to cleanse the treated area and their bodies every 3 days using a designated cleanser.

### 2.5 Outcome Measures

**FIGURE 1:**
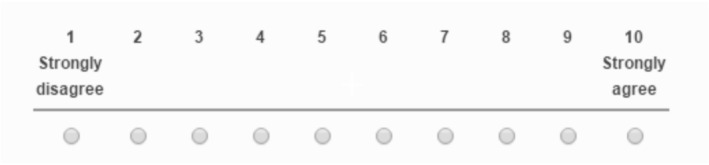
Ten‐point Likert scale.

Evaluation was performed by a blinded dermatologist at Days 10 and 30 post‐surgery using a 10‐point Likert scale [10]. The erythema score was based on the degree of redness, inflammation, and vascular response in the scar area, while the aesthetic score reflected overall visual quality, including pigmentation, texture, scar thickness, and uniformity. These measures were chosen due to their reliability in similar clinical studies and their ability to capture early changes in acute post‐surgical wounds. The evaluator remained blinded to treatment allocation throughout to ensure unbiased assessment (Figure 1).

### 2.6 Randomization and Blinding

To ensure the robustness and credibility of the findings, a randomization process was employed to assign the treatment and control conditions. Participants were randomly assigned to one of two groups using a computer‐generated randomization sequence. One breast of each participant received the topical treatment with timolol, while the contralateral breast was treated with saline as a control. This within‐subject design mitigated variability between participants and controlled for individual differences in scar formation.

Blinding was implemented at two levels: participant blinding and evaluator blinding. Participants were unaware of which breast received the timolol treatment and which received the saline to eliminate bias in reporting outcomes related to pain, aesthetics, or satisfaction with treatment. Similarly, a blinded dermatologist assessed the wound healing and scar appearance without knowing which treatment had been applied to each breast. This dual blinding approach aimed to minimize the potential for bias in both the participants' and evaluators' assessments, thereby strengthening the validity of the study outcomes.

### 2.7 Statistical Analysis

Data were collected and analyzed using SPSS software. Descriptive statistics were employed for initial data assessment, and *t*‐tests and ANOVA were utilized for quantitative variables. Chi‐square tests were applied for qualitative variables, and two‐way repeated measures ANOVA was used to compare the effects of timolol versus saline.

### 2.8 Ethical Considerations

This study received ethical approval from a recognized institutional review board (IRB) (Approval Code: IR.IUMS.FMD.REC.1401.555). All procedures were conducted in accordance with the principles of the Declaration of Helsinki and its subsequent revisions. Written informed consent was obtained from all participants after they were fully informed about the study's aims, procedures, potential risks, and benefits. Participation was voluntary, and participants had the right to withdraw at any time without consequences. All personal data were anonymized and securely stored to ensure confidentiality and compliance with ethical research standards.

## 3 Results

The evaluation of breast scar appearance concerning erythema was conducted by a dermatologist using a 10‐point Likert scale. Assessments were carried out on Days 2, 10, and 30 post‐surgery. The results are summarized in Table 1.

**TABLE 1:** Comparison of erythema levels in the patient's breast scar after mammoplasty, measured on a 10‐Point Likert scale (*t*‐test results and *p*‐values).

Erythema	Day 2	Day 10	Day 30
Placebo	6.75 ± 2	5.33 ± 2.05	4.08 ± 1.83
Timolol	6.91 ± 1.62	4.16 ± 1.19	2.16 ± 0.71
*p*‐value	0.825	0.1	0.003

**FIGURE 2:**
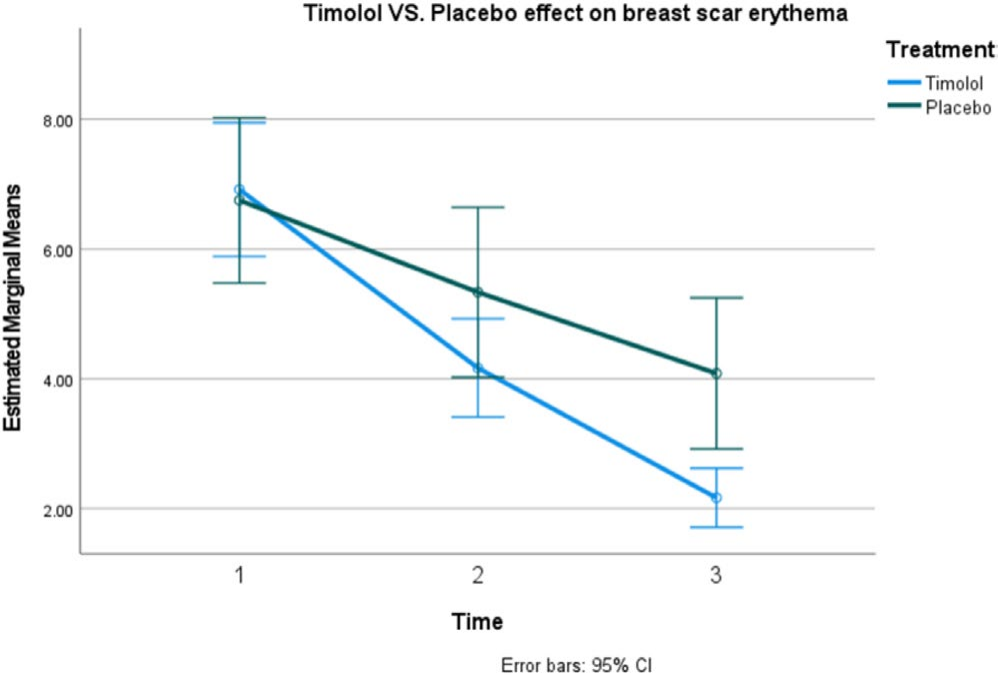
Comparison of the effectiveness of topical timolol versus placebo in reducing breast scar erythema.

**FIGURE 3:**
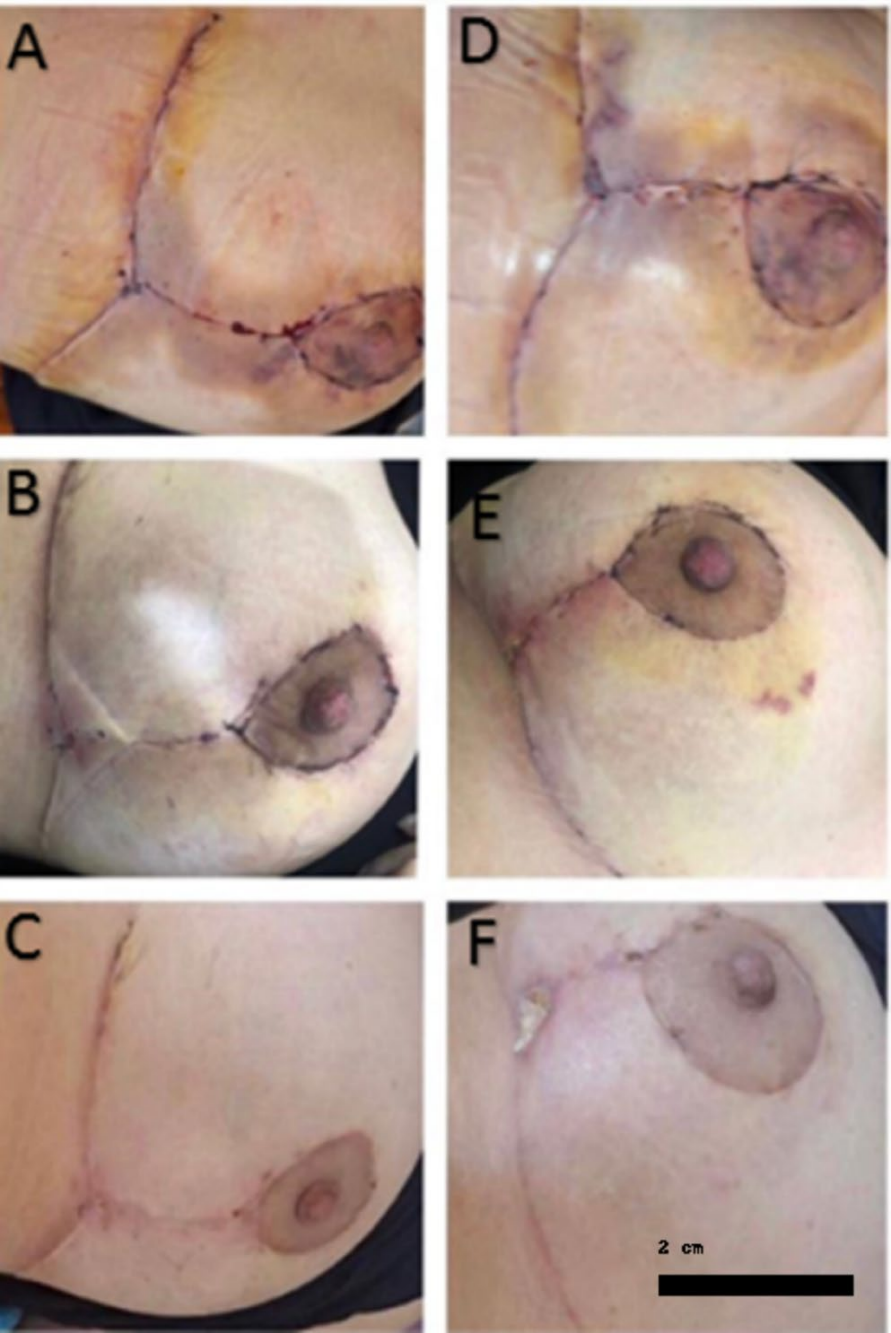
Comparison of erythema and aesthetic appearance between the timolol group and the control group. (A) Before intervention (2 days post‐surgery); (B) 10 days post distilled water; (C) 28 days post distilled water (30 days post‐surgery); (D) Before intervention; (E) 2 days post topical timolol; (F) 28 days post topical timolol.

**FIGURE 4:**
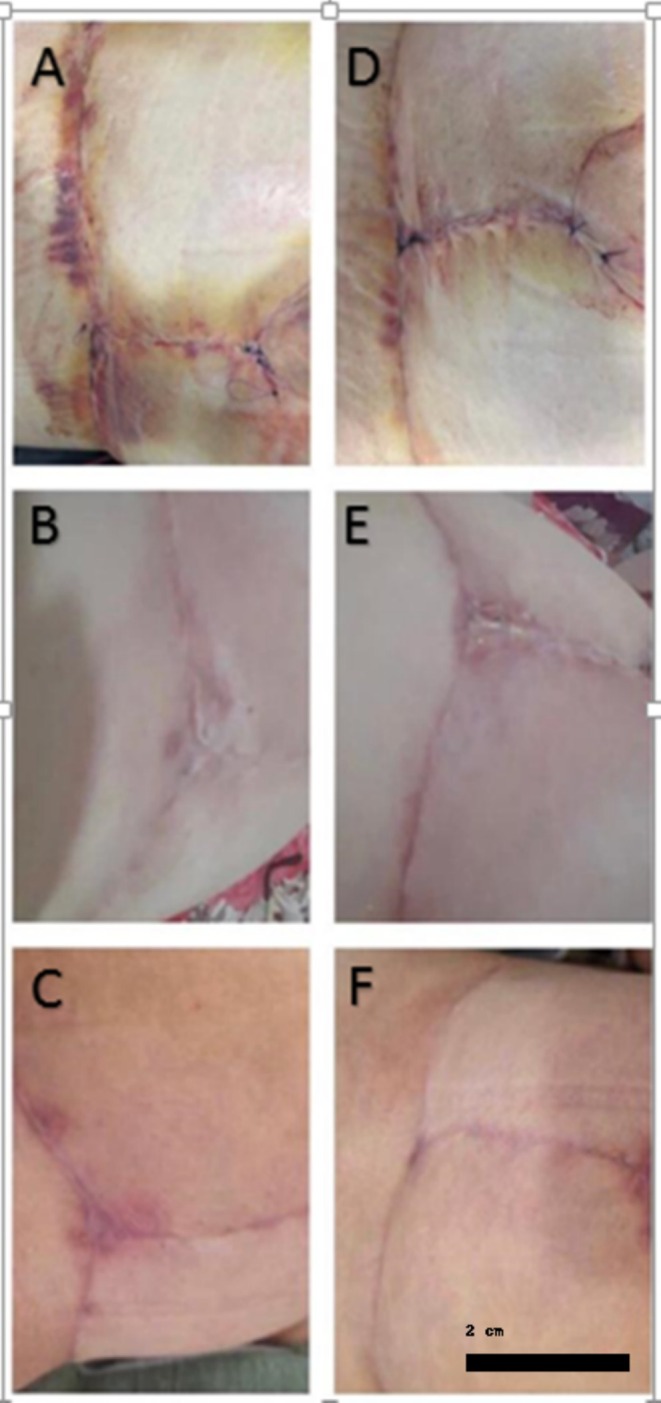
Comparison of erythema and aesthetic appearance between the timolol group and the control group. (A) Before intervention (2 days post‐surgery); (B) 10 days post distilled water; (C) 28 days post distilled water (30 days post‐surgery); (D) Before intervention; (E) 2 days post topical timolol; (F) 28 days post topical timolol.

Analysis utilizing two‐way repeated measures ANOVA revealed significant differences in erythema levels based on the type of medication administered over time (interaction, *p* < 0.0001; treatment, *p* = 0.02). Timolol resulted in a reduction of erythema by 5.38 points (95% CI: 4.22–6.55), while the placebo reduced erythema by 4.41 points (95% CI: 3.83–5). When comparing timolol to placebo, the topical timolol reduced erythema by an additional 0.972 points (95% CI: 0.18–1.7) over the course of 1 month (Figures 2, 3, 4).

The aesthetic appearance of the patient's breast scars was evaluated by a dermatologist using a 10‐point Likert scale on Days 2, 10, and 30. The results of this assessment are presented in Table 2.

**TABLE 2:** Comparison of aesthetic scores between control and timolol groups on days 2, 10, and 30 post‐surgery.

Aesthetic	Day 2	Day 10	Day 30
Placebo	2.91 ± 2.06	4.83 ± 2.08	6 ± 1.9
Timolol	2.66 ± 1.61	5.83 ± 1.52	8.1 ± 1.02
*p*‐value	0.744	0.193	0.002

**FIGURE 5:**
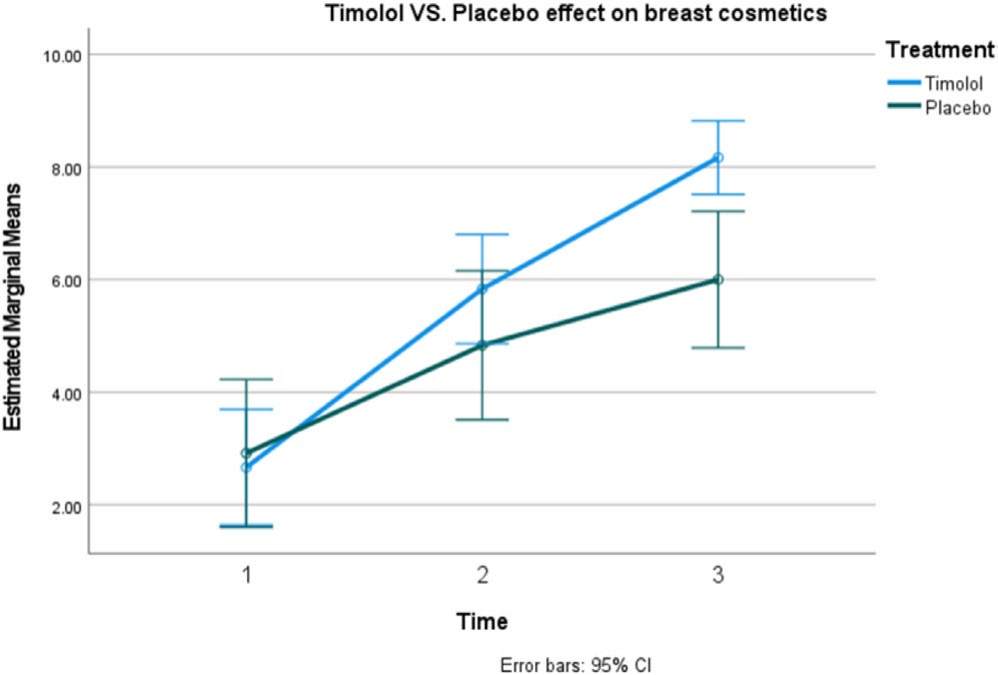
Effectiveness of topical timolol compared to placebo in improving the aesthetic appearance of breast scars.

The two‐way repeated measures ANOVA indicated significant differences in aesthetic scores based on the type of medication and the time of evaluation (interaction, *p* < 0.0001; treatment, *p* = 0.015). Timolol led to an increase in the aesthetic score of approximately 5.5 points over 1 month (95% CI: 4.9–6.2), while the placebo raised the aesthetic score by about 4.58 points (95% CI: 3.4–5.7). Overall, timolol improved the aesthetic score by 0.972 points more compared to placebo during the 1‐month period (95% CI: 0.23–1.7) (Figures 3, 4, 5).

## 4 Discussion

Timolol promotes the healing of both surgical and non‐surgical chronic wounds through multiple mechanisms. By inhibiting β1 and β2 adrenergic receptors expressed on keratinocytes, fibroblasts, and immune cells, timolol modulates the inflammatory response by regulating neutrophil and macrophage activity. It further enhances keratinocyte migration and proliferation through activation of the ERK and AKT signaling pathways and reduces fibrosis driven by fibroblasts. Additionally, timolol facilitates matrix remodeling via modulation of matrix metalloproteinases (MMPs) and supports angiogenesis, all of which contribute to accelerated wound healing and improved scar appearance [11, 12, 13, 14]. These effects align with previous studies demonstrating timolol's ability to increase healing speed and enhance overall wound quality compared to placebo [15, 16].

Numerous case reports on timolol and chronic wounds suggest favorable effects of this compound in wound healing. However, the doses used in these studies vary [17, 18, 19, 20]. One study evaluated the beneficial effects of a 0.1% gel on acute wounds [21], while our study employed a topical timolol concentration of 0.5%.

Statistical analyses showed that the type of intervention had a significant positive impact on aesthetic scores as assessed by a dermatologist. Specifically, during the 1‐month administration of timolol, this medication demonstrated up to 1 point better effects compared to placebo. This aligns with previous research by Manci and colleagues, which focused on the rate of re‐epithelialization, size, and thickness of wounds. Manci et al. demonstrated that after using 0.5% timolol for 4 weeks, wound re‐epithelialization occurred more rapidly and effectively, leading to a reduction in wound thickness [16]. Furthermore, the study by Beroukhim and colleagues on the healing of surgical scalp wounds treated with 0.5% timolol indicated that granulation tissue became flattened, and complete re‐epithelialization occurred by the end of 2 months [14]. Dabiri's study involving six patients post‐Mohs surgery for nonmelanoma skin lesions showed wound healing based on the VAS criteria after 5 weeks of timolol use compared to saline [15]. Notably, our study encompassed a larger sample size than previous studies and examined the effects of topical timolol from two aspects: erythema and aesthetics.

A study by Baltazard et al. in 2021 investigated the effect of timolol on venous leg ulcers (VLU). In this study, 43 patients were randomly selected, with 40 receiving at least one treatment and included in the analysis. At Week 12, a greater than 40% reduction in ulcer size was observed in 14 out of 21 patients (67%) treated with timolol, compared to 6 out of 19 patients (32%) in the control group. Importantly, no serious side effects were reported, similar to our findings [12].

In a 2020 study by Rai et al., the effects of topical timolol on chronic venous ulcers were examined. A total of 20 patients meeting the inclusion and exclusion criteria were enrolled, all having chronic venous ulcers. The outcome was evaluated based on the reduction in ulcer area. The mean reduction in ulcer area for patients treated with timolol dressings was 86.80%, compared to 43.82% in the saline dressing group. Notably, complete wound closure occurred in five patients (50%) in the timolol group, whereas no complete wound closures were observed in the saline group [13].

The results of the study conducted by Aya Reda Mohamed Hawwas and colleagues in 2023 show that the use of Timolol Maleate 0.5% after fractional carbon dioxide laser treatment, compared to the use of fractional CO_2_ laser alone, has a significant impact on the improvement of atrophic acne scars. Both treatments showed significant improvement after treatment; however, no significant difference in the degree of improvement between the two groups was observed. The overall conclusion is that the application of timolol after CO_2_ laser treatment can provide comparable improvement, and due to its high safety profile, easy accessibility, low cost, and non‐invasive nature, it could be a suitable option for treating acne scars. However, further research with larger sample sizes and controlled conditions is needed to confirm these results [22].

The results of the study conducted by Ghanbarzamani and colleagues in 2021 demonstrated the efficacy and safety of topical 0.25% Timolol Gel (TG) in promoting wound healing in split‐thickness skin graft (STSG) donor sites. The study, a double‐blind, randomized clinical trial, revealed that the use of timolol significantly accelerated re‐epithelialization, reducing healing time to 11.5 ± 2.3 days, compared to 14.5 ± 3.2 days in the placebo group. Additionally, patients in the timolol group experienced significantly less pain, as evidenced by lower visual analog scale (VAS) scores on Days 1 through 7. Furthermore, there was no incidence of infection in either group, and no transplant rejection was observed in the timolol‐treated group. The Vancouver scar scale (VSS) scores at 3 months showed a significant improvement in the timolol group, indicating better scar quality. These findings suggest that Timolol Gel can be a promising, cost‐effective treatment option for enhancing wound healing and reducing pain in patients with skin grafts [23].

The study conducted by Dabiri and colleagues in 2017 investigated the effect of topical timolol on improving the overall cosmesis of acute surgical wounds. The research aimed to assess if timolol, a nonselective β‐adrenergic receptor antagonist, could enhance the appearance of wounds after excision or Mohs micrographic surgery (MMS) for nonmelanoma skin cancers. The study involved six participants who were randomly assigned to either the timolol treatment group or the saline (placebo) group. The results showed that wounds treated with timolol had significantly better cosmetic outcomes, as assessed by the Visual Analog Scale (VAS), than those treated with saline. The VAS score for the timolol‐treated group was 6.5 ± 0.9, while the control group scored 2.5 ± 0.7, indicating a notably better cosmetic result in terms of pigmentation, vascularity, acceptability, and contour. The findings suggest that topical timolol may be an effective option to improve the cosmesis of acute surgical wounds, providing an innovative approach to wound care for dermatological procedures [15].

In addition to post‐surgical scars, timolol has been successfully used in the treatment of chronic venous ulcers, hypertrophic scars, and donor sites of skin grafts. It has demonstrated efficacy in promoting re‐epithelialization, reducing erythema, and minimizing scar hypertrophy. These dermatological uses further support the potential of timolol as a versatile and non‐invasive topical treatment for wound management [12, 13, 14, 17, 21].

### 4.1 Limitations

This study has several limitations that should be considered when interpreting the findings. First, the small sample size (12 participants) may limit the statistical power and generalizability of the results. Second, the follow‐up period was limited to 4 weeks, which may not be sufficient to assess long‐term scar maturation or recurrence. Third, outcome assessments were performed using subjective Likert scale ratings by a blinded dermatologist, which, although common in clinical studies, may introduce variability; objective measures such as 3D imaging, dermoscopy, or digital erythema quantification could provide more robust evaluations. Additionally, the strict exclusion criteria (e.g., patients with comorbidities, smokers, or those with skin disorders) reduce the external validity of the findings. Future studies should aim to address these limitations by employing larger, more diverse cohorts, incorporating objective imaging and histological assessments, and extending the follow‐up duration to better capture the long‐term efficacy and safety of topical timolol in scar management.

## 5 Conclusion

This randomized controlled trial demonstrated that topical 0.5% timolol significantly improves the healing quality of immature post‐surgical scars following bilateral mammoplasty. Compared to saline, timolol led to greater reductions in erythema and better aesthetic outcomes, with statistically significant improvements observed over a 1‐month period. These findings support the use of timolol as a promising non‐invasive option for enhancing early scar healing. Despite the study's limitations—such as a small sample size and relatively short follow‐up—the consistent effects observed warrant further investigation in larger, long‐term trials. Future research should also explore the underlying molecular mechanisms and evaluate the generalizability of these results across different surgical contexts and patient populations.

## Author Contributions

N.N. and A.G. designed the study. A.J., F.N., A.J., and E.B. wrote the paper. S.T.R., S.S., N.H., and M.R. edited the manuscript. All authors have read and approved the content of the manuscript.

## Disclosure

Transparency declaration: Authors declare that the manuscript is honest, accurate, and transparent. No important aspect of the study is omitted.

## Ethics Statement

The researchers were committed and adhered to the principles of the Helsinki Convention and the Ethics Committee of Iran University of Medical Sciences in all stages.

## Conflicts of Interest

The authors declare no conflicts of interest.

## Data Availability

The data that support the findings of this study are available from the corresponding author upon reasonable request.
